# STING-Mediated Interferon Induction by Herpes Simplex Virus 1 Requires the Protein Tyrosine Kinase Syk

**DOI:** 10.1128/mbio.03228-21

**Published:** 2021-12-21

**Authors:** Chenyao Wang, Nikhil Sharma, Manoj Veleeparambil, Patricia M. Kessler, Belinda Willard, Ganes C. Sen

**Affiliations:** a Department of Inflammation and Immunity, Lerner Research Institute, Cleveland Clinic, Cleveland, Ohio, USA; Duke University Medical Center

**Keywords:** HSV-1, STING signaling, Tyr phosphorylation, Syk, EGFR, interferon

## Abstract

The nature and the intensity of innate immune response to virus infection determine the course of pathogenesis in the host. Among the many pathogen-associated molecular pattern recognition receptors, STING, an endoplasmic reticulum (ER)-associated protein, plays a pivotal role in triggering responses to microbial or cellular cytoplasmic DNA. Herpes simplex virus 1 (HSV-1), a common human pathogen, activates STING signaling, and the resultant induction of type I interferon causes inhibition of virus replication. In this context, we have observed that phosphorylation of Tyr245 of STING by epidermal growth factor receptor kinase is necessary for interferon induction. Here, we report that phosphorylation of Tyr240 by the tyrosine kinase Syk is essential for all signaling activities of STING. Our analysis showed that upon ligand-binding, STING dimerizes and interacts with membrane-bound EGFR, which autophosphorylates and provides the platform for the recruitment of cytoplasmic Syk to the signaling complex and its activation. Activated Syk phosphorylates Tyr240 of STING, followed by phosphorylation of Tyr245 by epidermal growth factor receptor (EGFR). Pharmacological or genetic ablation of Syk activity resulted in an arrest of STING in the ER compartment and a complete block of gene induction. Consequently, in the absence of Syk, HSV-1 could not induce interferon, and it replicated more robustly.

## INTRODUCTION

STING plays a critical role in eliciting beneficial cellular responses to a variety of diseases, including microbial infection, cancer, inflammation, and autoimmune diseases (1–7). Therefore, a better understanding of the various steps of STING signaling and their regulation will lead to better management and treatment of important health problems. The best-known function of STING is its role as an adaptor protein on which signaling complexes assemble and activate transcription factors, such as IRF3 and NF-κB, to induce the synthesis of various cytokines, including type I interferon (IFN). The ligands of STING are the various 2′,3′-linked cyclic dinucleotides produced by intracellular bacteria or cGAMP synthesized by the mammalian cytoplasmic enzyme cGAS, which is activated by cellular or microbial DNA if present in the cytoplasm (8–10). Many DNA viruses, such as herpes simplex virus 1 (HSV-1), trigger STING signaling, presumably due to leakage of viral DNA from the nucleus to the cytoplasm (11, 12). STING-mediated induction of IFN inhibits HSV-1 replication, and in the absence of this negative feedback loop, the virus replicates better and is more pathogenic in mice (13, 14).

STING resides in the endoplasmic reticulum (ER) membrane, but its cytoplasmic domain carries out all of its known functions, including ligand binding and signaling. A unique feature of STING signaling is that it is accompanied by translocation of the protein from the ER to various other intracellular membrane compartments (15, 16). It transits from the ER to the ERGIC (ER-Golgi intermediate compartment) to travel further by two alternative routes: it can go to the Golgi body and then to late endosomes, or it can go to autophagophores and autophagosomes to trigger autophagy. The transcriptional signaling via NF-κB occurs when STING is in the ERGIC or the ER, whereas IRF3 activation requires its presence in the late endosomes (6, 16, 17). STING functions as a dimer, a process that is facilitated by its binding to another adaptor protein, TRIF (18). Ligand binding to a well-defined pocket of STING changes its conformation and initiates the signaling process (19). In the early steps of signaling, STING undergoes several posttranslational modifications (20, 21). We discovered that ligand-induced phosphorylation of Tyr 245 of STING is essential for its ability to signal by the IRF3, but not the NF-κB, branch of signaling (16). We identified ER-bound epidermal growth factor receptor (EGFR) as the tyrosine kinase responsible for STING Tyr 245 phosphorylation, which is required for IRF3 activation and type I IFN induction (16). Without Tyr245 phosphorylation by EGFR, activated STING cannot translocate to the late endosomes, where it binds IRF3 and triggers its activation through its phosphorylation by STING-bound TBK1 (16).

Here, we report that phosphorylation of another tyrosine residue of human STING, Tyr 240 (Tyr 239 in mice), is required for both NF-κB and IRF3 activation. In the absence of this phosphorylation, activated STING could not transit from the ER to the ERGIC. Moreover, we identified the protein tyrosine kinase Syk as the enzyme that phosphorylates Tyr 240 of STING. Syk is a nonreceptor tyrosine kinase that contains two Src homology 2 (SH2) domains, which mediate its interaction with phosphotyrosine residues of other proteins (22, 23). Syk is well known for its role in B cell functions, and although it is highly expressed in hematopoietic cells, it is also expressed in a wide variety of other cells (22, 24, 25). Syk is located in the cytoplasm and can bind to the plasma membrane through lipid modifications (26). We demonstrated that Syk is recruited to STING by binding to EGFR. Our analyses allowed us to delineate new steps of STING signaling, mediated by EGFR and Syk. As expected, HSV-1 replication was augmented in the absence of Syk, in a STING-dependent fashion.

## RESULTS

### Syk kinase activity is required for gene induction by STING signaling.

**FIG 1:**
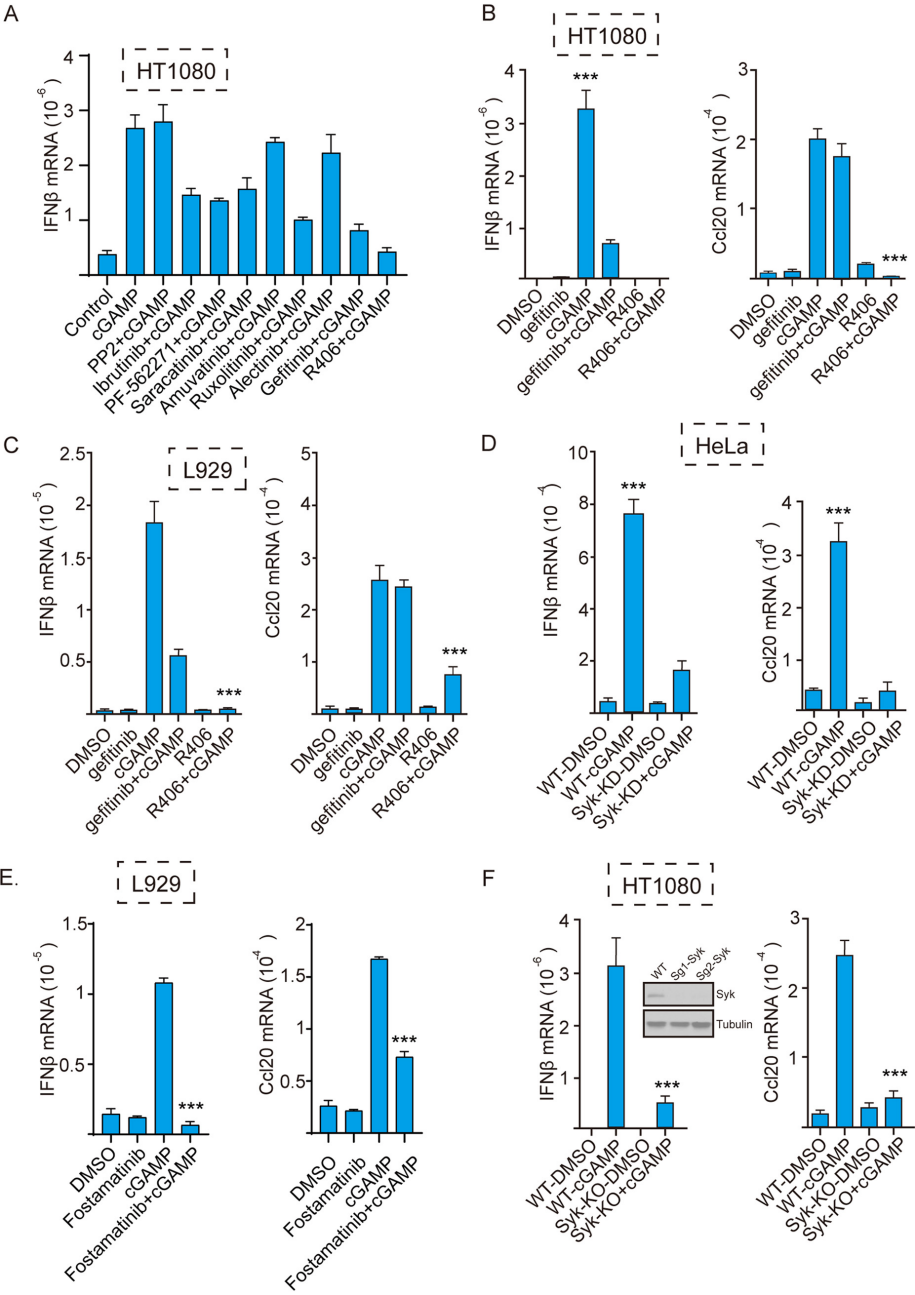
Syk kinase activity is required for gene induction by cGAMP-activated STING. (A) Different protein tyrosine kinase inhibitors were tested for their ability to inhibit STING-mediated gene induction; cells were pretreated with the inhibitor before cGAMP stimulation. The inhibitors were PP2 (Src inhibitor; 20 μM), ibrutinib (BTK inhibitor; 10 μM), PF-562271 (FAK inhibitor; 20 μM), saracatinib (Fyn inhibitor; 10 μM), amuvatinib (c-Kit inhibitor; 10 μM), ruxolitinib (Jak1/2 inhibitor; 10 μM), alectinib (ALK inhibitor; 10 μM), gefitinib (EGFR inhibitor; 10 μM), and R406 (Syk inhibitor; 10 μM). (B and C) The Syk inhibitor R406 inhibited STING-mediated induction of IFN-β and Ccl20 mRNAs in (B) human HT 1080 and (C) mouse L929 cells. Cells were pretreated with dimethyl sulfoxide (DMSO), gefitinib, or the Syk inhibitor R406 (10 μM) before cGAMP transfection; after 5 h, mRNAs were measured (*n* = 3). Bars show means and standard deviations (SD) for 3 independent experiments. ***, *P* < 0.001 compared to the R406+cGAMP-treated group by two‐tailed Student’s *t* test. (D) Syk KD HeLa cells were treated with cGAMP; after 5 h, Ccl20 and IFN-β mRNAs were measured (*n* = 3). Bars show means and SD for 3 independent experiments. ***, *P* < 0.001 compared to the Syk-KD+cGAMP group. (E) The Syk inhibitor fostamatinib (10 μM) inhibited STING-mediated induction of IFN-β and Ccl20 mRNAs in L929 cells. (F) Syk KO HT1080 cells were treated with cGAMP; after 5 h, Ccl20 and IFN‐β mRNAs were measured (*n* = 3). The Syk KO effect were verified through WB using Syk antibody; two clonal lines were tested (inset). Bars show means and SD for 3 independent experiments. ***, *P* < 0.001 compared to the Syk KO+cGAMP group.

To identify the protein tyrosine kinases that are required for gene induction by STING signaling, we screened the effects of known inhibitors of different families of tyrosine kinases on STING-mediated IFN-β induction. R406, an inhibitor of Syk, strongly blocked STING-mediated gene induction (Fig. 1A). In both human HT1080 cells (Fig. 1B) and mouse L929 cells (Fig. 1C), IFN-β mRNA induction by cyclic GMP-AMP (cGAMP)-activated STING was strongly inhibited by both R406 and gefitinib, an inhibitor of EGFR (27), indicating that both Syk and EGFR are required for STING signaling. IFN-β is induced by the transcription factors IRF3 and NF-κB, whereas induction of Ccl20 mRNA requires only NF-κB. As reported before, gefitinib did not inhibit Ccl20 mRNA induction, but R406 inhibited induction of both IRF3-driven and NF-κB-driven genes. Thus, there was a distinct difference between the mechanisms of involvement of the two kinases in STING signaling. The need of Syk for STING signaling was confirmed further using human HeLa cells in which the Syk gene had been knocked down (Fig. 1D). In addition, another inhibitor of Syk, fostamatinib, inhibited the induction of IRF3-driven and NF-κB-driven genes (Fig. 1E). Finally, we knocked out the Syk gene in HT1080 cells using CRISPR/Cas9 technology; several clonal cell lines did not express any Syk (Fig. 1F, inset). When one of these lines was tested, STING activation failed to induce either IFN-β mRNA or Ccl20 mRNA.

### Syk is required for STING-mediated inhibition of HSV-1 replication.

**FIG 2:**
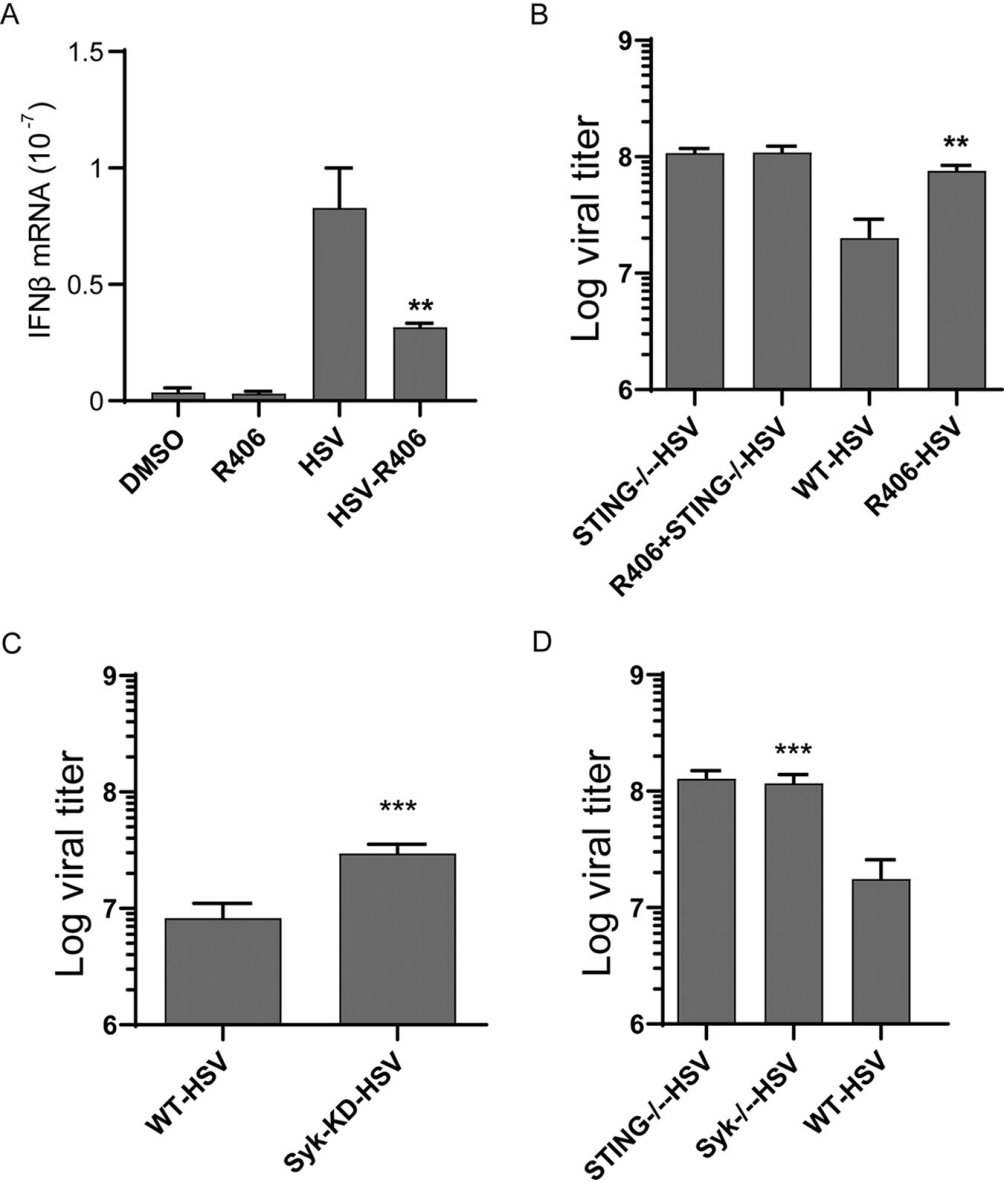
STING-mediated IFN induction and its antiviral effect on HSV-1 replication require Syk. (A) HT1080 cells were pretreated with R406 for 1 h and infected with HSV-1, RNA was extracted at 4 h postinfection, and IFN-β mRNA induction was measured (*n* = 3). Bars show means and SD for 3 independent experiments. **, *P* < 0.01 compared to the HSV-infected group by two-tailed Student’s *t* test. (B) HT1080 and HT1080 STING^−/−^ cells were pretreated with the Syk inhibitor R406 followed by HSV-1 infection, and viral titers were determined by plaque assays at 16 h postinfection (MOI = 5). Bars show means and SD from 3 independent experiments. **, *P* < 0.01 compared to the HSV-infected WT group by two-tailed Student’s *t* test. (C) WT HeLa cells and Syk KD cells were infected with HSV-1 (MOI = 5), and viral titers were determined by plaque assays at 16 h postinfection Bars show means and SD from 3 independent experiments. ***, *P* < 0.001 compared to the HSV-infected WT group by two-tailed Student’s *t* test. (D) WT HT1080 cells, STING^−/−^ HT1080 cells, and Syk^−/−^ cells were infected with HSV-1 (MOI = 5), and viral titers were determined by plaque assays at 16 h postinfection. Bars show means and SD from 3 independent experiments. ***, *P* < 0.001 compared to the HSV-infected WT group by two-tailed Student’s *t* test.

HSV-1 infection triggers STING signaling, and the resultant induction of IFN causes inhibition of virus replication. We used this system to test the need of Syk for STING signaling in a physiological context (Fig. 2). IFN induction by HSV-1 was inhibited by the Syk inhibitor R406 (Fig. 2A). Consequently, virus replication was augmented in the inhibitor-treated cells (Fig. 2B); however, the Syk inhibitor had no effect on virus replication in STING^−/−^ cells, indicating that it regulated virus replication only through inhibition of STING signaling. Finally, the need of Syk for inhibiting HSV-1 replication was confirmed using Syk knockdown (KD) HeLa cells (Fig. 2C) and Syk^−/−^ (knockout [KO]) HT1080 cells (Fig. 2D).

### Syk promotes STING signaling by phosphorylating its Tyr 240 residue.

**FIG 3:**
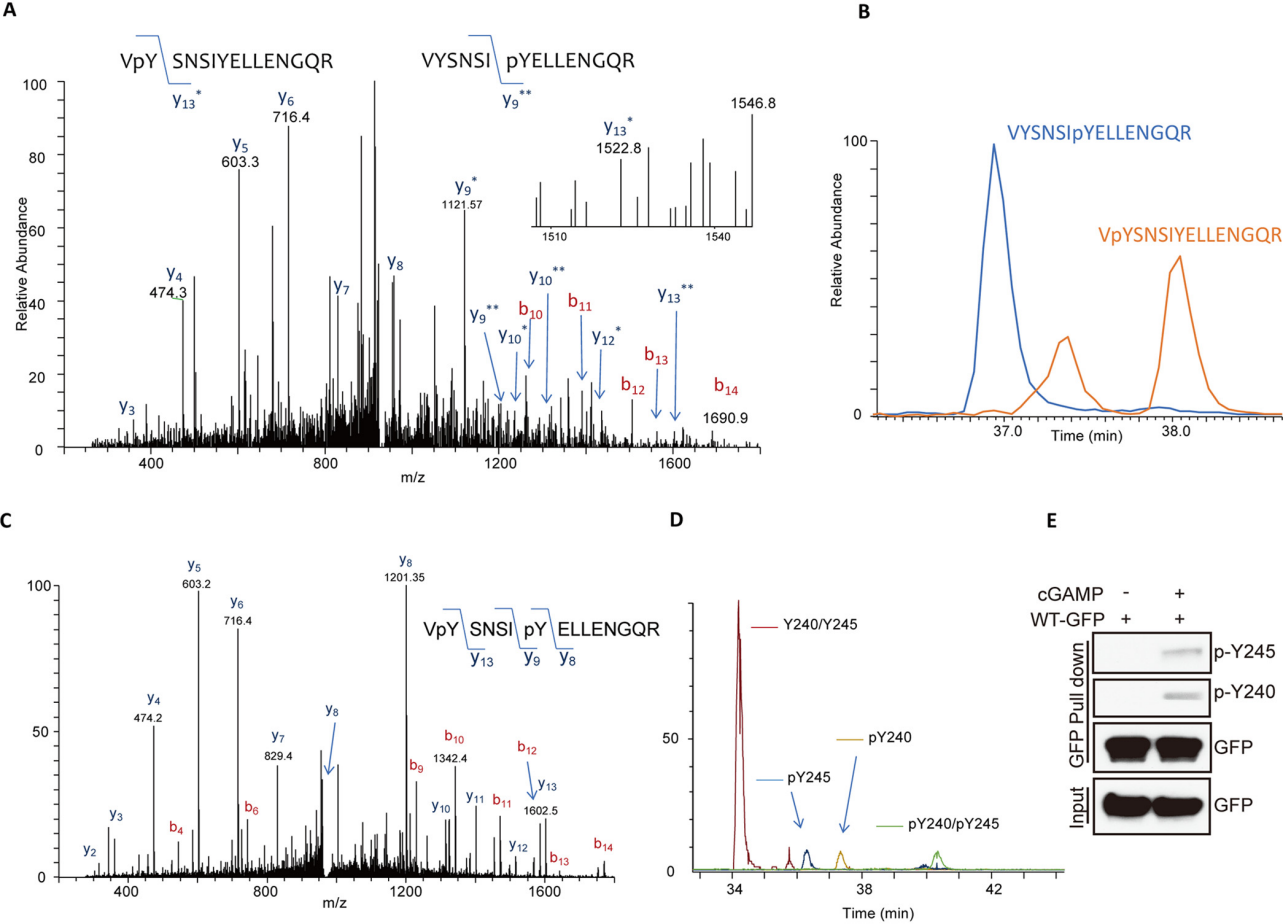
Tyr240 of STING is phosphorylated upon cGAMP stimulation. (A) After 3 h of cGAMP stimulation, STING-GFP was purified from 293XL cells via GFP trap beads, fractionated on an SDS-PAGE gel, and digested with trypsin and chymotrypsin, and the digests were analyzed by LC-MS/MS. A doubly charged peptide with a mass of 932.93 Da was identified in a targeted analysis of GFP-STING. The collision-induced dissociation (CID) spectra for this peptide contains several C-terminal y ions, and the masses of these ions are consistent with the presence of more than one phosphoisoform. The mass difference of the y13* peptide is consistent with modification at Y240, and the mass of the y9** peptide is consistent with modification at Y245. (B) The degree of modification from A was determined by plotting chromatograms for both the unmodified and modified forms of the Y240/Y245 peptides. The chromatograms for the VYSNSIpYELLENGQR and VpYSNSIYELLENGQR peptides are shown. (C) A doubly charged peptide with a mass of 972.911 Da was identified in a targeted analysis of STING-GFP. The CID spectrum for this peptide contains several C-terminal y ions, and the masses of the y8, y9, and y13 ions are consistent with phosphorylation at Y240 and Y245. (D) Chromatograms for the unmodified, pY240, pY245, and pY240-pY245 peptides from STING-GFP WT. The phosphopeptides are several orders of magnitude lower in abundance than the unmodified peptide. (E) Specific antibodies detecting pY240 and pY245 phosphorylation of STING in cGAMP treated 293 XL cells expressing STING-GFP.

After confirming the need of Syk for STING signaling, we set out to investigate its mechanistic basis. In view of the fact that phosphorylation of Y245 of STING by EGFR is needed for IFN induction (16, 28), we inquired whether another Tyr residue of STING is phosphorylated upon stimulation and whether Syk is required for the process. For this purpose, STING-green fluorescent protein (GFP)-expressing cells were stimulated with cGAMP; STING-GFP was purified from the cell extract and subjected to liquid chromatography-tandem mass spectrometry (LC-MS/MS) analysis after protease digestion. Two isoforms of a phosphopeptide spanning V239 to R253 were identified; the mass of one was consistent with modification at Y240 and that of the other with modification of Y245 (Fig. 3A). The degree of modification was determined by plotting chromatograms of both modified and unmodified forms of the peptide (Fig. 3B). A doubly phosphorylated form of the same peptide was identified as well (Fig. 3C). The abundance of singly or doubly phosphorylated peptides was low compared to that of the unmodified peptide (Fig. 3D), possibly because only a small portion of the highly expressed STING-GFP was engaged in signaling. For the sake of facilitating detection of Y240 or Y245 phosphorylation of STING, we raised antibodies to the two respective phosphopeptides. As expected, these antibodies did not recognize STING-GFP pulled down from extracts of untreated cells, but both reacted with it if cells were stimulated with cGAMP (Fig. 3E). These phosphospecific antibodies were extensively used for many experiments, as described below.

**FIG 4:**
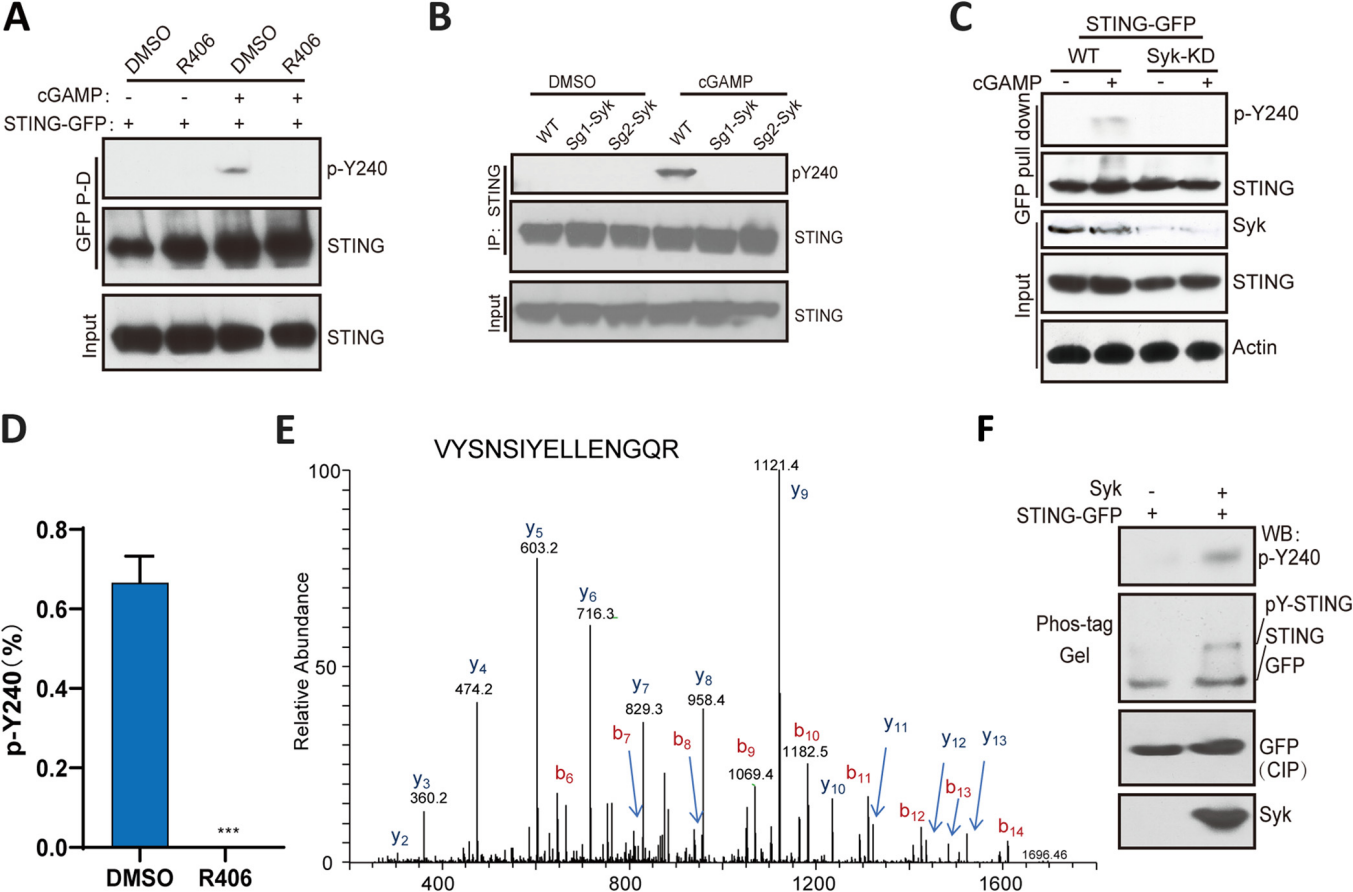
Syk phosphorylates Tyr240. (A) R406 inhibits the phosphorylation of STING at Y240. STING-GFP transfected HT1080 STING^−/−^ cells were treated with R406 for 1 h, followed by 3 h of cGAMP treatment, the lysates were subjected to GFP pulldown and analyzed with pY240-specific antibodies. (B) In Syk KO HT1080 cells, there was no detectable pY240 after cGAMP stimulation. WT and Syk-KO HT1080 cells were treated with cGAMP, and the lysates were subjected to IP via STING antibody and Western blotting with pY240 specific antibodies. (C) In Syk KD HeLa cells, there was no detectable pY240 even after cGAMP stimulation. WT and Syk-KD HeLa cells were transfected with STING-GFP, followed by the treatment with cGAMP. The lysates were subjected to GFP pulldown and Western blotting with pY240 specific antibodies. (D) LC-MS/MS analysis showing inhibition of phosphorylation of Y240 by the Syk inhibitor. STING‐GFP-293XL cells were pretreated with R406 and then treated with cGAMP. LC-MS/MS analysis was done, as described above, for detecting pY240 (*n* = 3). Bars show means and SD from 3 independent experiments. ***, *P* < 0.001 compared to R406 pretreated group by two‐tailed Student’s *t* test. (E) LC-MS/MS of STING from R406-pretreated and cGAMP (3 h)-transfected 293XL cells. A doubly charged peptide with a mass of 892.946 Da was identified in a targeted analysis of STING-GFP. The CID spectra for this peptide contain several C-terminal y ions, and the masses of these ions are consistent with the unmodified VYSNSIYELLENGQR peptide. (F) Syk phosphorylates STING on Y240 *in vitro*. Purified STING‐GFP from 293XL cells was incubated with commercial Syk for *in vitro* kinase assays; the products were analyzed on Phos‐tag gel by Western blotting using pY240-specific antibody and GFP antibody (top two panels). The proteins were analyzed, before assay incubation, with the indicated antibodies (bottom two panels).

We have previously reported that Y245 is phosphorylated by STING-bound EGFR (16). Because of our observation that Syk was required for gene induction by STING, we inquired whether Syk phosphorylated Y240. Western blotting with the antibody against the Y240 phosphopeptide revealed that the Syk inhibitor R406 blocked Y240 phosphorylation (Fig. 4A). Moreover, there was no phosphorylation of this residue in Syk KO (Fig. 4B) and Syk KD cells (Fig. 4C). These results were confirmed by mass-spectrometric analysis: no pY240-containing phosphopeptide was detectable in STING-GFP isolated from R406-treated cGAMP-stimulated cells (Fig. 4D and E). These results indicated that Syk activity was required for Y240 phosphorylation. To determine whether Syk could directly phosphorylate Y240, purified STING-GFP was incubated with Syk in an *in vitro* kinase assay. Western blotting with the pY240-specific antibody showed direct phosphorylation of this residue by Syk (Fig. 4F, top); analysis on phospho-tag gel, which separates phosphorylated proteins from the unphosphorylated form, confirmed this conclusion (Fig. 4F, Phos-tag gel).

### Increasing phosphorylation of STING Y240 leads to increasing gene induction.

**FIG 5:**
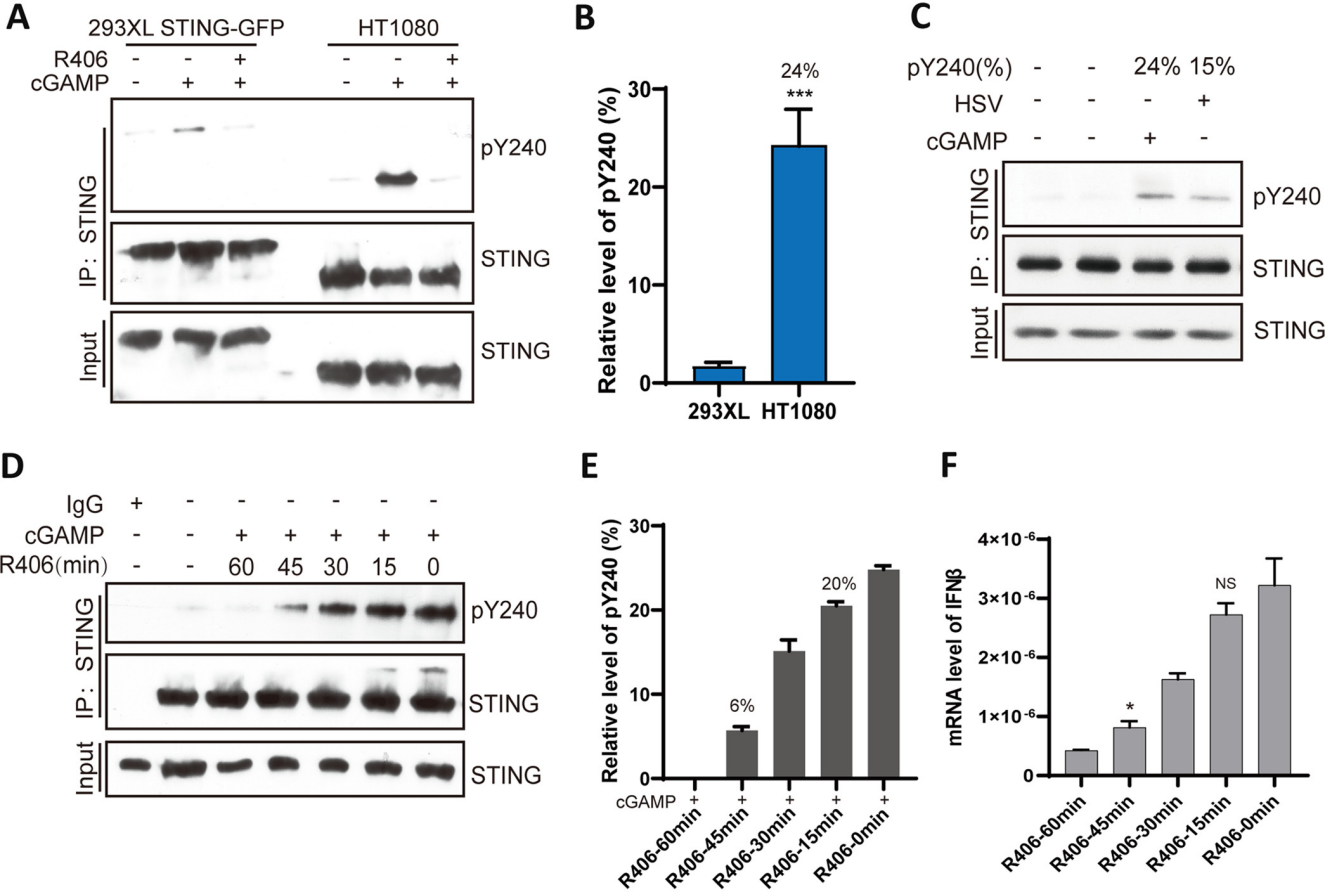
Gene induction by activated STING parallels its Y240 phosphorylation status. (A) Comparison of Y240 phosphorylation in 293XL STING-GFP and HT1080 cells. In order to display similar levels of STING, 5 times more HT1080 cells were used in these experiments; cells were subjected to 3 h of cGAMP treatment followed with IP with STING antibody and Western blotting with pY240 specific antibody. (B) Twenty-four percent of STING was phosphorylated on Y240 in HT1080 cells and 0.7% in 293XL cells (*n* = 3). ***, *P* < 0.001 compared to the R406-pretreated group by two‐tailed Student’s *t* test. (C) After 3 h of HSV infection, 15% of STING was phosphorylated on Y240. (D) The phosphorylation of STING at pY240 decreases with an increase in the time of pretreatment with R406. After pretreatment with R406, HT1080 cells were transfected with cGAMP followed with IP and WB detection via pY240 specific antibody 3 h posttransfection. (E) The pY240 of STING was quantified from results in panel D (*n* = 3). (F) HT1080 cells were pretreated with R406 for the indicated times, followed by 5 h cGAMP treatment, and IFN-β mRNA levels were measured (*n* = 3). Bars show means and SD from 3 independent experiments. *, *P* < 0.05. NS, not significant compared with the non-R406-pretreated group by two‐tailed Student’s *t* test.

The mass-spectrometric analysis of Y240 phosphorylation in 293XL cells, in which STING-GFP was overexpressed to facilitate its purification, showed that only 0.7% of this residue was phosphorylated after stimulation (Fig. 4). To estimate the level of Y240 phosphorylation of endogenous STING in HT1080 cells, we used the pY240 antibody in Western blot analysis of immunoprecipitated STING from different cell extracts. When similar levels of STING (or STING-GFP) were precipitated from HT1080 and 293XL cells, we observed stronger phosphorylation of Y240 upon cGAMP stimulation of HT1080 cells (Fig. 5A); compared to 0.7% phosphorylation of STING Y240 in 293XL cells, 24% was phosphorylated in HT1080 cells (Fig. 5B), and in HSV-infected cells, the corresponding number was 15% (Fig. 5C). To determine whether increasing Y240 phosphorylation causes increasing STING signaling, we pretreated HT1080 cells for different time periods with the Syk inhibitor R406, before cGAMP stimulation. Increasing lengths of treatment caused decreasing Y240 phosphorylation (Fig. 5D). More importantly, gene induction levels (Fig. 5F) paralleled the levels of Y240 phosphorylation (Fig. 5E), plateauing at around 20% phosphorylation. These results demonstrated a quantitative relationship between Y240 phosphorylation and gene induction by STING.

### Syk activity is required for translocation of STING from the ER to the ERGIC.

**FIG 6:**
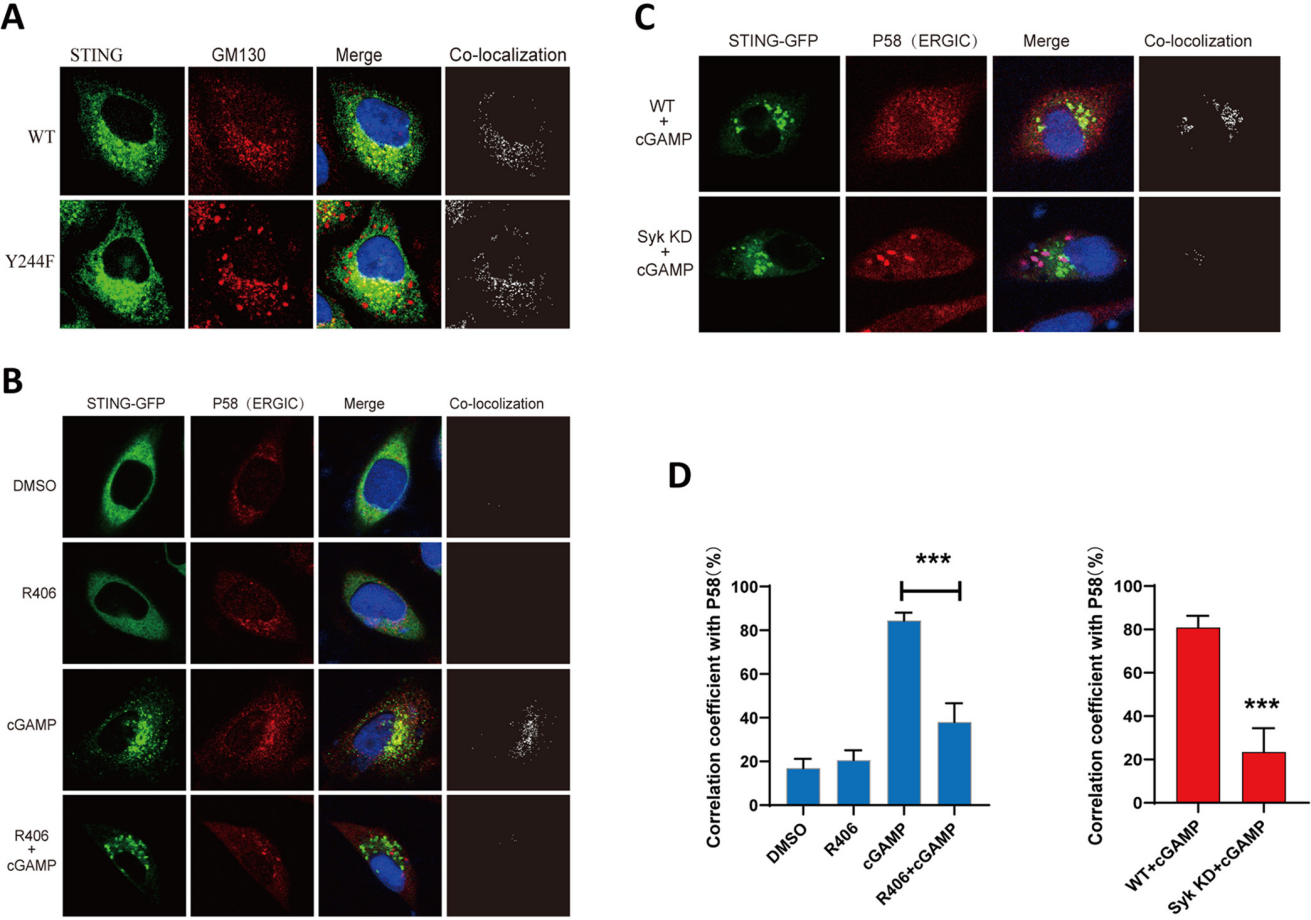
Syk is needed for STING translocation from the ER to the ERGIC. (A) The Y244F mutant goes to the Golgi body after cGAMP treatment. STING^−/−^ MEF cells were transfected with WT STING-GFP and the Y244F mutant (green) for 24 h and then transfected with cGAMP for 2.5 h; cells were fixed and labeled with GM130 (Golgi body; red) antibody. (B) STING translocation to the ERGIC requires Syk activity. STING^−/−^ MEF cells were transfected with STING-GFP (green) for 24 h, pretreated with R406 for 1 h, and transfected with cGAMP for 2.5 h; cells were fixed and labeled with P58 (ERGIC; red) antibody. (C) Syk is needed for STING trafficking to ERGIC. Syk KD HeLa cells were transfected with STING-GFP (green) for 24 h, pretreated with R406 for 1 h, and transfected with cGAMP for 2.5 h; cells were fixed and labeled with P58 (ERGIC; red) antibody. (D) Colocalization coefficient between the STING-GFP and P58 in the indicated cells. At least 30 cells were quantified from each independent experiment, which was repeated three times with similar results. Values are means and SD. ***, *P* < 0.001.

STING signaling is accompanied by its translocation from the ER compartment to other membranous compartments, and different branches of signals emanate from different intracellular organelles. For example, IRF3 activation, which is needed for IFN-β induction, requires transit of STING to the late endosomes; a process that requires phosphorylation of Y245 (Y244 in mice). We have reported that in the absence of phosphorylation of this residue by EGFR, which takes place in the ER, STING does not translocate to the late endosome, although it leaves the ER and enters the ERGIC, an intermediate stop between the ER and the Golgi bodies (16). Further analysis by confocal microscopy showed that, after cell activation by cGAMP, the Y244F mutant of STING could travel to the Golgi bodies as well (Fig. 6A), reducing the need of Y244 (245) phosphorylation for the transit of STING between the Golgi and the endosomes. We proceeded to seek similar information for the effect of Y240 phosphorylation by Syk on STING translocation. However, we could not use the Y240F mutant of STING for these analyses, because Y240 resides in the ligand-binding pocket of STING and its mutation inhibits cGAMP binding (19, 29). Instead, we evaluated the need of Syk for STING translocation. Confocal analysis showed that either the presence of R406 (Fig. 6B) or the absence of Syk (Fig. 6D) prevented the transit of STING from the ER to the ERGIC. Colocalization of STING-GFP and P58, a marker for the ERGIC, was quantitated in multiple cells, in the presence or the absence of EGFR activity, to confirm the above conclusion (Fig. 6C).

### Syk activation and early steps of STING signaling.

**FIG 7:**
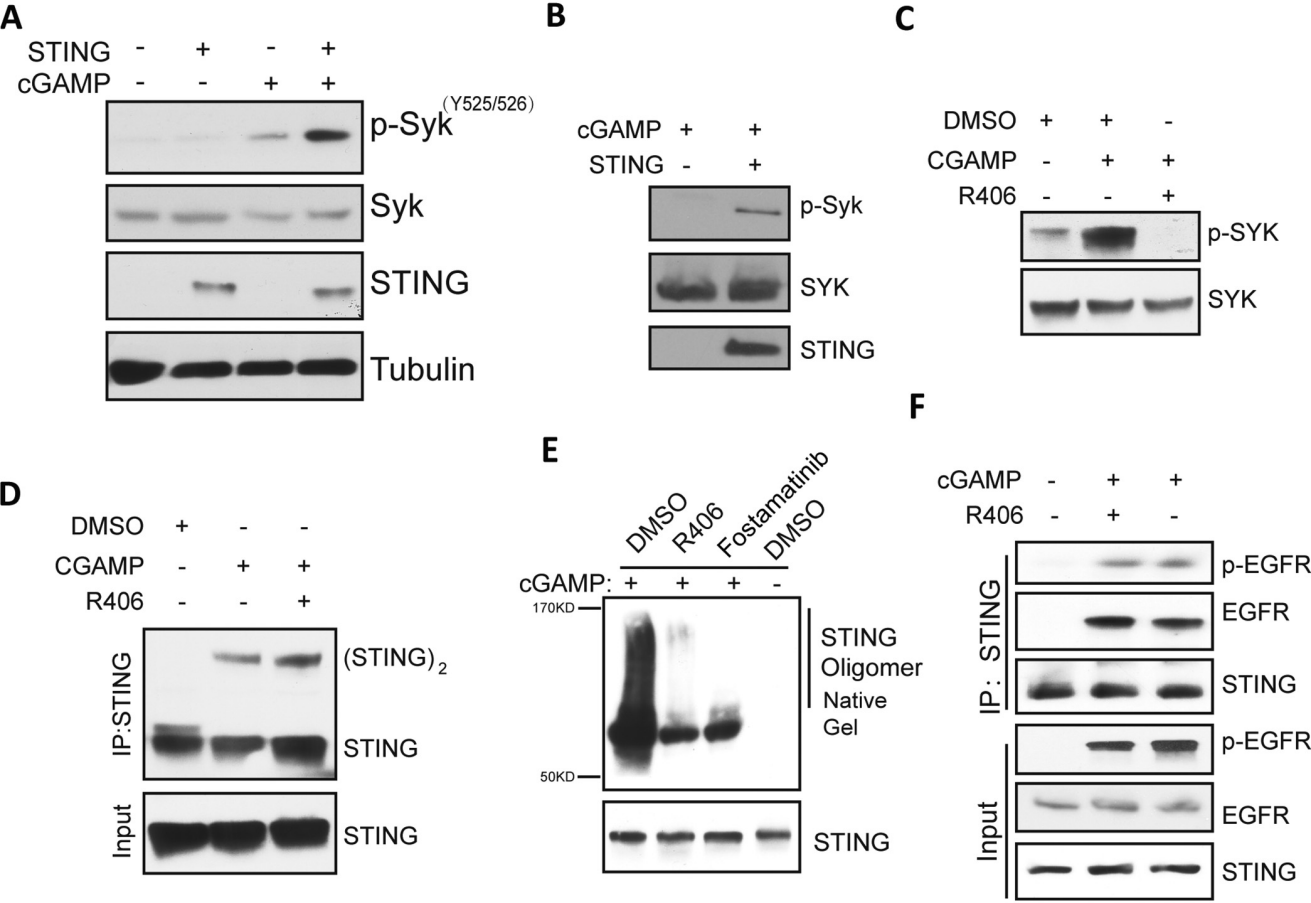
cGAMP-stimulated Syk activation requires STING and Syk kinase activity. (A) Syk activation in a cGAMP-STING-dependent manner. HT1080 cells were treated with cGAMP for 1 h, and the cell lysates were subjected to WB with the indicated antibody to Syk pY525/526. (B) cGAMP-induced Syk activation is STING dependent. MEF WT and STING^−/−^ cells were treated with cGAMP for 1 h, and the cell lysates were subjected to WB with the antibody to Syk pY525/526. (C) R406 prevents cGAMP-induced Syk activation. HT1080 cells were pretreated with R406 followed by cGAMP treatment for 1 h, and the cell lysates were subjected to WB with the antibody to Syk pY525/526. (D) Syk is not needed for STING dimerization. HT1080 cells were pretreated with R406 for 1 h and then transfected with cGAMP for 3 h; after IP with STING antibody, the proteins were analyzed by electrophoresis on a nonreducing gel and detected with STING antibody. (E) Syk activity is required for oligomerization of STING. HT1080 cells were pretreated with the indicated Syk inhibitor, followed by 4 h of cGAMP treatment. The cell lysates were subjected to nondenaturing PAGE and WB analysis using STING antibody. (F) Syk inhibitor does not affect the activation of EGFR. HT1080 cells were pretreated with R406 for 1 h and treated with cGAMP for 1 h, and the cell lysates were subjected to IP with STING antibody and WB with the pY1068 antibody of EGFR.

The above results indicated that Syk was required for one or more early steps of STING signaling that take place within the ER. For activation of the protein kinase activity of Syk, it needs to be phosphorylated on Tyr525/526 (30). This activation required cGAMP stimulation of cells and the presence of STING (Fig. 7A and B). Moreover, inhibiting Syk kinase activity by its inhibitor, R406, prevented Syk phosphorylation (Fig. 7C), indicating that it is autophosphorylated upon binding to STING, which is known to dimerize when it binds to its ligand. STING dimerization is the first step of the signaling cascade, and it did not require Syk activity (Fig. 7D). However, Syk inhibitors greatly attenuated STING oligomerization (Fig. 7E), which happens after STING exits the ER compartment (15). Another early step of this signaling pathway is activation of EGFR by binding to STING (16); this step also did not require Syk activity (Fig. 7F).

### Both Syk and EGFR kinases are needed for phosphorylation of Tyr240 and Tyr245 of STING.

**FIG 8:**
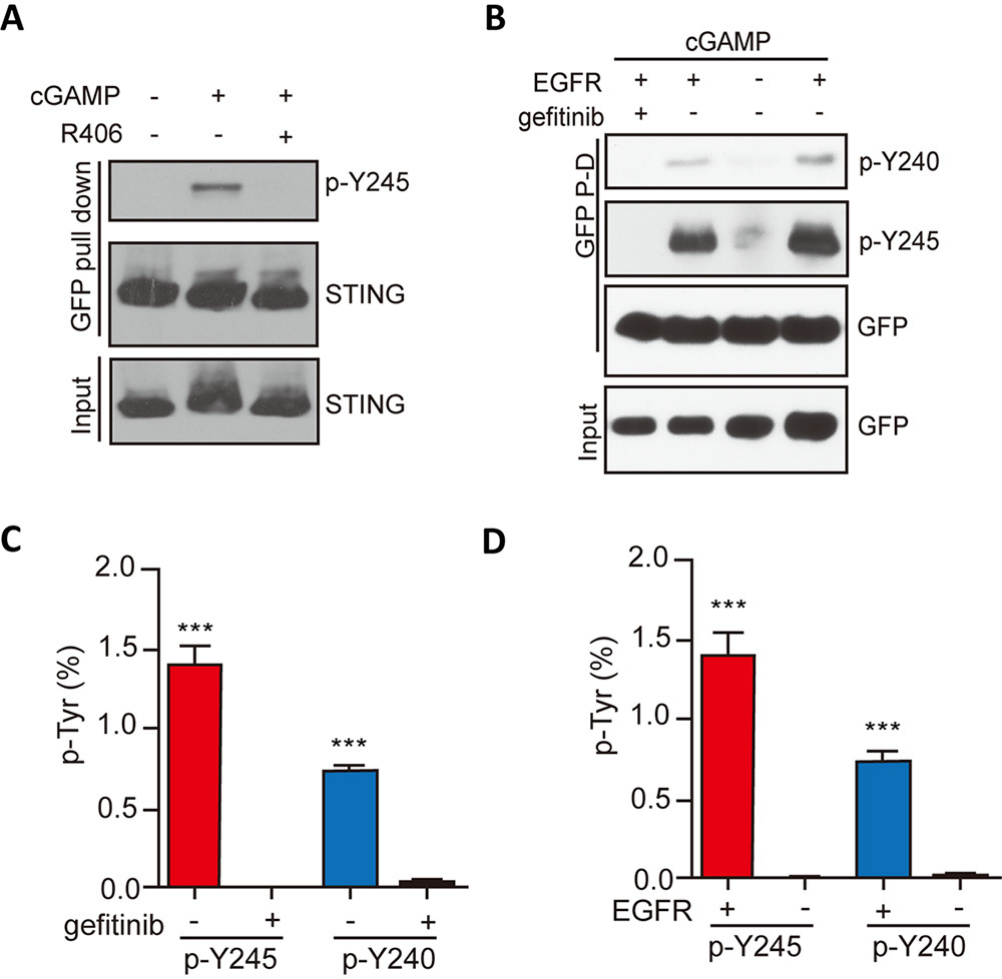
Both Syk and EGFR are needed for phosphorylation of Tyr240 and Tyr245. (A) Syk activity is required for Y245 phosphorylation. HT1080 cells were pretreated with R406 for 1 h and treated with cGAMP for 3 h, and the cell lysates were subjected to WB with the pY245 antibody of STING. (B) Gefitinib and EGFR KO inhibited both Y245 and Y240 phosphorylations. (C) Gefitinib inhibited the phosphorylation of Y240 and Y245, as revealed by LC-MS/MS analysis. Bars show means and SD from 3 independent LC-MS/MS experiments. ***, *P* < 0.001 compared to the gefitinib‐pretreated group by two‐tailed Student’s *t* test. (D) The absence of EGFR inhibited the phosphorylation of both Y240 and Y245. STING phosphorylation was analyzed by LC‐MS/MS as described above. Bars show means and SD from 3 independent LC-MS/MS experiments. ***, *P* < 0.001 compared to the EGFR KD group by two‐tailed Student’s *t* test.

**FIG 9:**
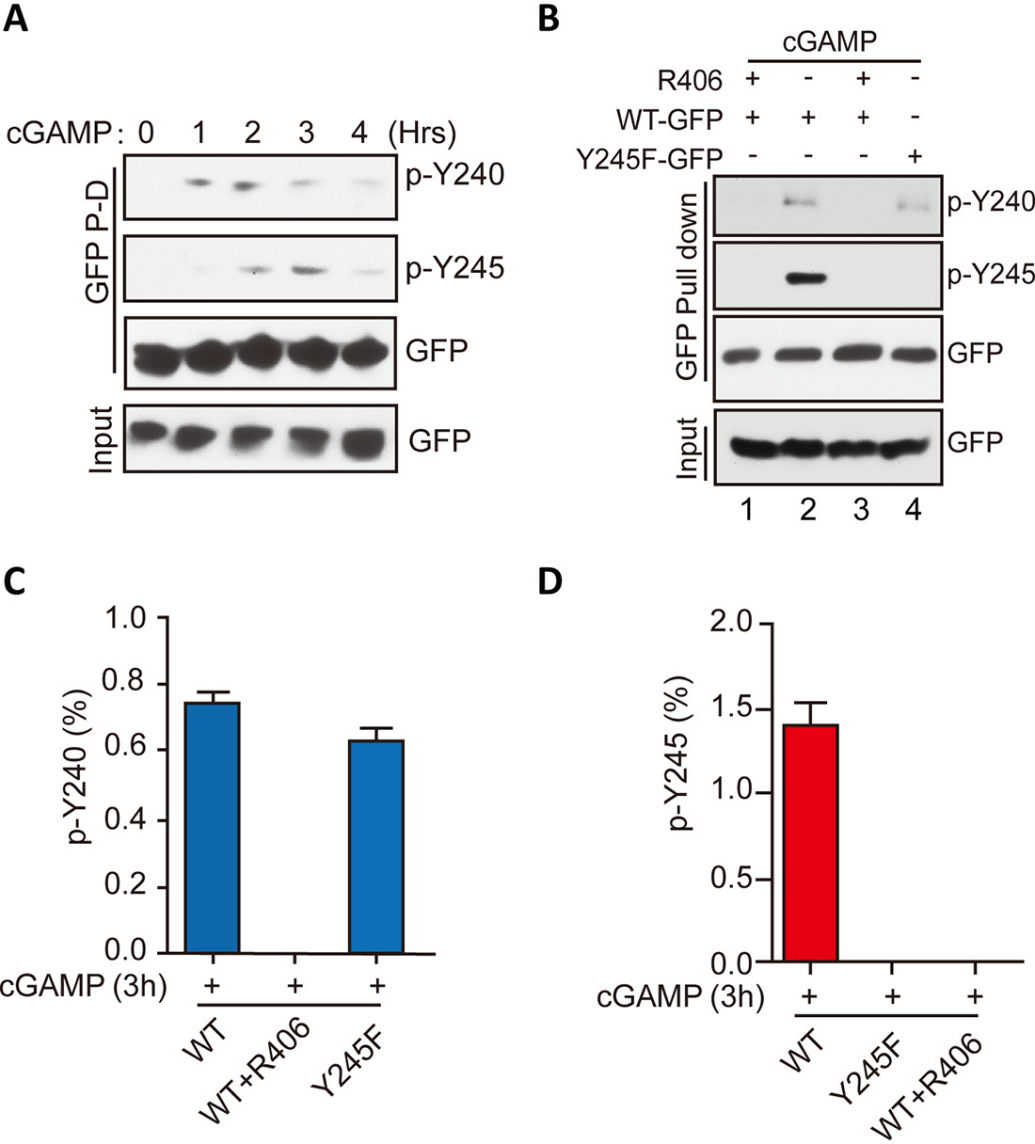
Tyr245 phosphorylation requires prior phosphorylation of Tyr240. (A) Kinetics of STING phosphorylation after cGAMP stimulation. The cell lysates were subjected to GFP pulldown and WB with antibodies to pY240 and pY245. (B) Y240 phosphorylation was needed for Y245 phosphorylation, but not vice versa. (C) The Y245F mutant still showed pY240, as revealed by LC-MS/MS analysis. Bars show means and SD from 3 independent LC-MS/MS experiments. (D) Without Syk activity, there was no pY245. Bars show means and SD from 3 independent LC-MS/MS experiments.

Although *in vitro*, Y245 was directly phosphorylated by EGFR and Y240 was directly phosphorylated by Syk, we wondered whether, in cells, they were interdependent in triggering the signaling pathway; indeed, that was the case. Syk kinase activity was required for phosphorylating not only Y240 (Fig. 4) but also Y245 (Fig. 8A). The need of EGFR kinase activity for phosphorylation of either Y245 or Y240 of STING was determined using the site-specific antibodies (Fig. 8B) or LC-MS/MS analyses (Fig. 8C and D). When EGFR kinase activity was inhibited by gefitinib or EGFR^−/−^ cells were used, phosphorylation of both sites was abolished, indicating that EGFR activity was required, directly or indirectly, for cGAMP-stimulated modification of both Y245 and Y240. Kinetic analysis revealed that Y240 phosphorylation preceded Y245 phosphorylation (Fig. 9A). Moreover, Y240 of the Y245F mutant was phosphorylated (Fig. 9B, lane 4), indicating that Y240 phosphorylation requires both kinases but not Y245. Our conclusions were further supported by LC-MS/MS analyses of phosphorylation of the two Tyr residues of WT and Y245F STING (Fig. 9C and D).

### STING, EGFR, and Syk physically interact in the signaling complex.

**FIG 10:**
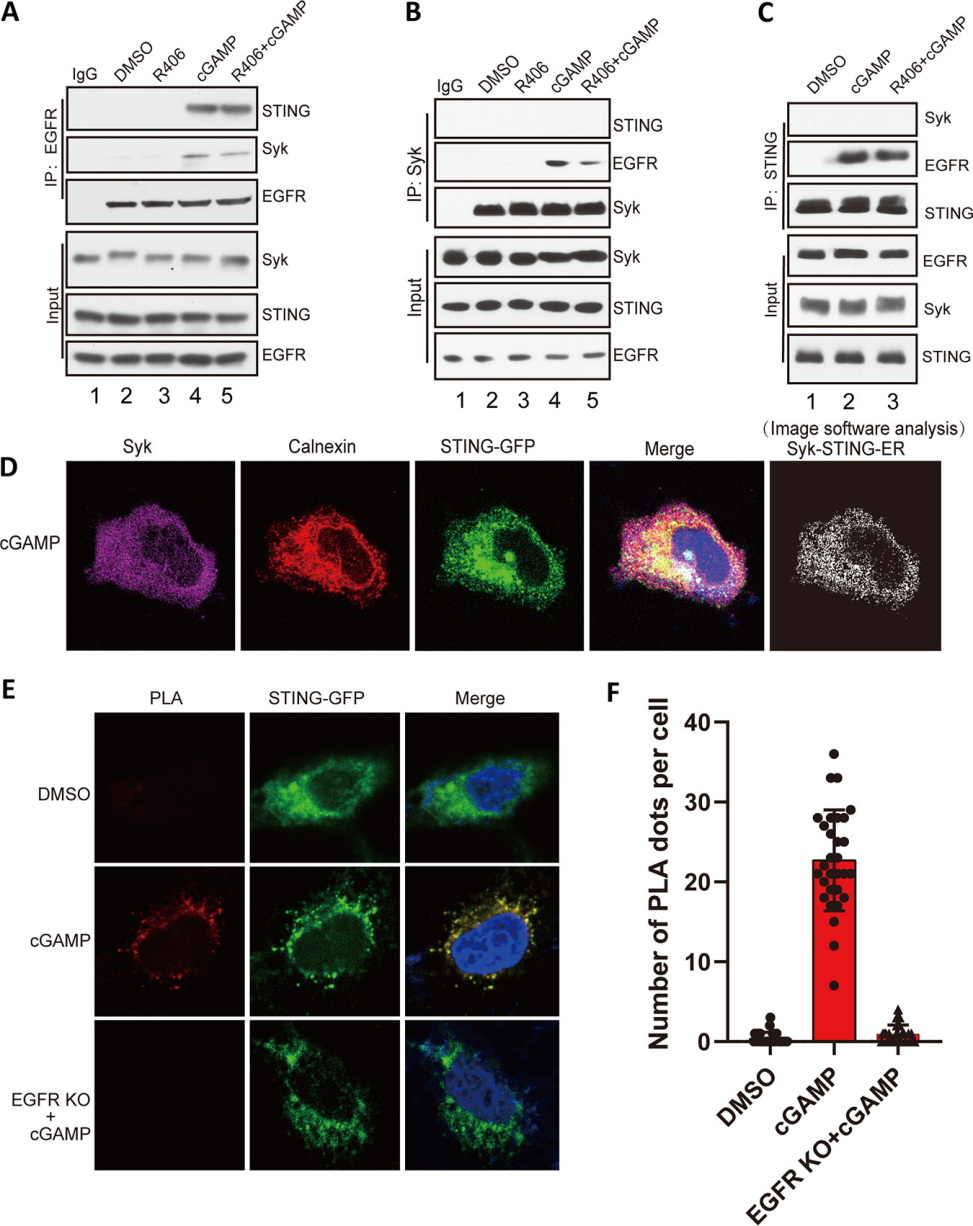
STING, EGFR, and Syk physically interact in the signaling complex. (A) EGFR interacts with Syk and STING upon cGAMP treatment, and this association does not depend on Syk activity. HT1080 cells were pretreated with R406 for 1 h and treated with cGAMP for 1 h, and the cell lysates were subjected to IP with EGFR antibody and analyzed by WB with Syk, STING, and EGFR antibodies. (B) Syk interacts with EGFR upon cGAMP treatment but not STING. HT1080 cells were pretreated with R406 for 1 h and treated with cGAMP for 1 h, and the cell lysates were subjected to IP with anti-Syk and analyzed via WB with the indicated antibodies. (C) STING binds to EGFR, but not Syk, upon cGAMP treatment. HT1080 cells were pretreated with R406 for 1 h and treated with cGAMP for 1 h, and the cell lysates were subjected to IP with anti-STING and analyzed via WB with the indicated antibodies. (D) Confocal experiment showed that STING and Syk colocalized on ER. HeLa cells were transfected with mGST-Syk and STING-GFP plasmids, treated with cGAMP for 1 h, fixed, and stained with calnexin (red) and GST antibody (purple). The white dots show STING-Syk colocalization on ER as revealed by Fuji analysis. (E) PLA technology showed that STING and Syk interacted in stimulated cells. The red dots show that STING and Syk were in close proximity. HeLa cells or HeLa EGFR KO cells were transfected with STING-GFP for 24 h, treated with cGAMP for 1 h, fixed, and stained with GFP and Syk antibody followed by PLA. (F) Quantification of PLA dots per cell from panel E (means and SD). *P* < 0.001. At least 30 cells were quantified from each independent experiment, which was repeated three times with similar results.

The results presented above showed that STING phosphorylation by EGFR and Syk was essential for its ability to signal. We set out to determine whether the two kinases physically associate with STING in the signaling complexes. We used coimmunoprecipitation assays using cell extracts and cell-based proximity ligation assays (PLA) for this purpose. When EGFR was immunoprecipitated from cGAMP-treated cells, both STING and Syk were associated with it (Fig. 10A, lane 4) and Syk activity was not required for these interactions (Fig. 10A, lane 5). When Syk was precipitated, EGFR came along with it (Fig. 10B, lane 4), but STING was not detectable; again, Syk activity was not required for Syk-EGFR interaction (Fig. 10B, lane 5). Similarly, precipitated STING was bound to EGFR (Fig. 10C, lane 2) even in R406-treated cells (Fig. 10C, lane 3), but Syk was not detectable. These results demonstrated EGFR interaction with both STING and Syk, but STING/Syk interaction was not apparent. We resorted to confocal microscopy to demonstrate colocalization of STING and Syk in the ER of stimulated cells (Fig. 10D). Finally, PLA showed that STING and Syk interacted in stimulated cells, but only if EGFR was present (Fig. 10E and F). These results indicated that EGFR bridges STING and Syk in the signaling complex.

## DISCUSSION

**FIG 11:**
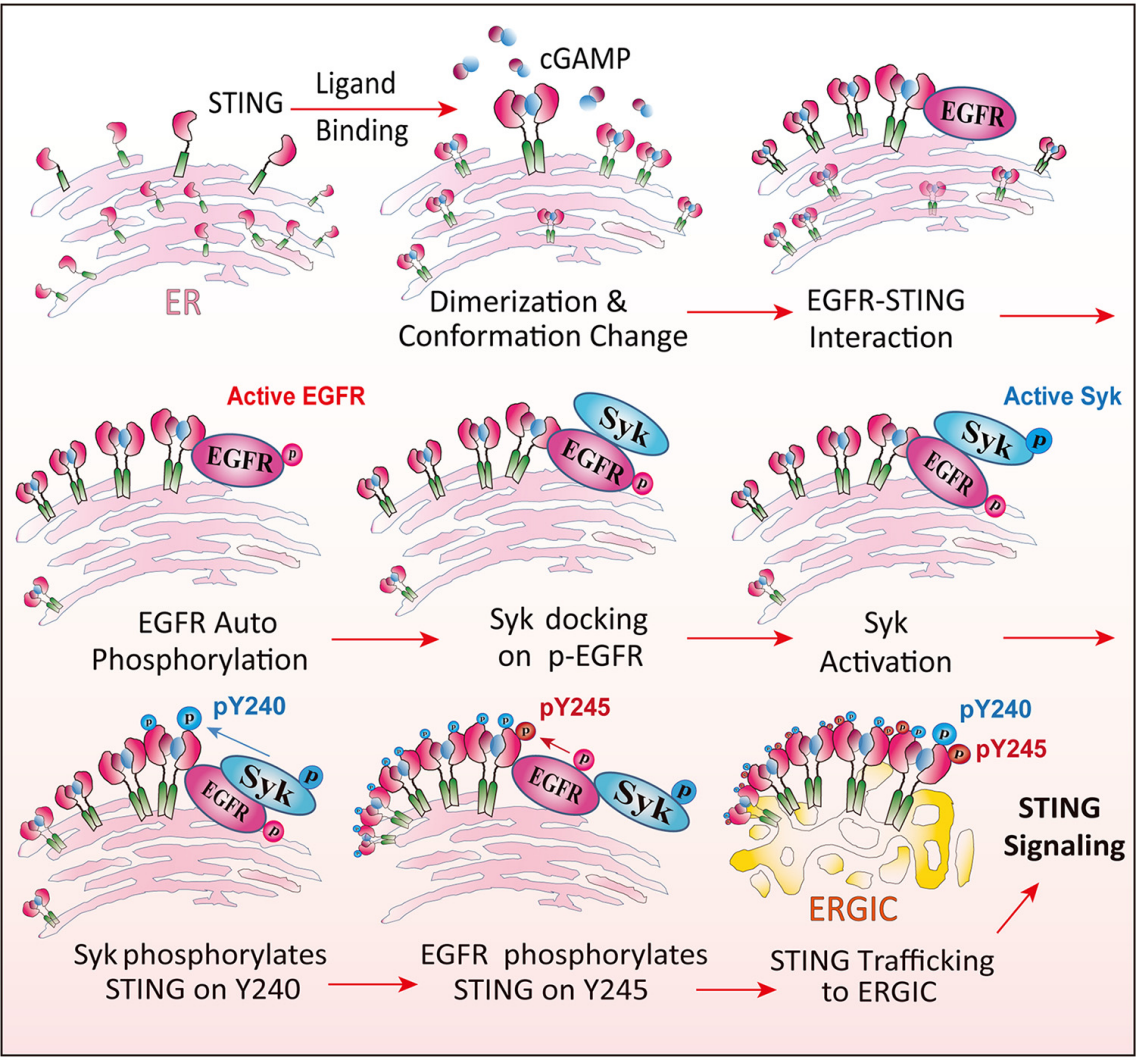
Steps of STING signaling in the ER. cGAMP binds to STING, causes its conformation change, and stabilizes STING dimers and oligomers. STING interacts with ER membrane-bound EGFR; EGFR then autophosphorylates, and Syk docks on p-EGFR and is activated. Syk phosphorylates STING on Y240; EGFR phosphorylates STING on Y245, and STING translocates to the ERGIC for signaling transduction.

The results presented above, regarding the early events of STING signaling, allow us to delineate the successive steps of the activation process leading to its translocation from the ER to the ERGIC (Fig. 11). It is known that cytoplasmic ligands, such as cGAMP, bind to the binding pocket of the protein, stabilize its dimeric form and change its conformation (31). We reported previously that activated STING recruits ER-bound EGFR, which is activated by autophosphorylation, possibly due to an enzyme-substrate interaction between two EGFR molecules bound to the two subunits of dimerized STING (16). The pTyr residue (Tyr 1068) of EGFR possibly served as the docking site for Syk, an SH2 domain-containing cytoplasmic protein. We presented evidence here that Syk was not needed for STING dimerization or EGFR activation (Fig. 7D and F). However, STING oligomerization, which occurs after STING transits from the ER, required Syk activity (Fig. 7E); this observation was consistent with our finding that in the absence of Syk, activated Syk did not exit from the ER (Fig. 6).

In the next step, Syk was activated by its autophosphorylation, a process that required STING activation and both EGFR and Syk enzyme activities (Fig. 7B and C). Activated Syk phosphorylated Tyr240 of STING, which did not require Tyr245 phosphorylation (Fig. 8B to D); the latter phosphorylation, however, required prior phosphorylation of Tyr240 by Syk (Fig. 8A). The doubly phosphorylated STING was fully active in signaling, a process that was accompanied by its trafficking to ERGIC (Fig. 6). Because Syk kinase activity was required for phosphorylation of both Tyr residues of STING, in its absence, STING did not exit the ER (Fig. 6B). The ER-bound STING could not induce either IRF3-driven or NF-κB-driven genes, indicating that its transit to the ERGIC might be necessary for any transcriptional signaling. The Y245F mutant of STING, on the other hand, could travel up to the Golgi bodies and induce NF-κB-driven genes as competently as the wild-type (WT) protein (Fig. 6A). We could not test the signaling property of the reciprocal mutant of STING, the Y240F mutant, because this mutation affects ligand binding and the mutant could not dimerize even after stimulation of the cells with cGAMP (data not shown).

The sequential actions of the two kinases, EGFR and Syk, on STING indicated that the three proteins physically interact as components of the signaling complex. We confirmed this interaction using two complementary approaches. Coimmunoprecipitation experiments demonstrated STING-EGFR and Syk-EGFR interactions, both of which required cGAMP stimulation of STING, implicating activated STING as the platform for assembly of the signaling complex (Fig. 10). Proximity ligation assays were used to demonstrate STING-Syk interaction. The lack of their coimmunoprecipitation could be due to the lability of the complex or its susceptibility to the salt concentrations of the extraction or the immunoprecipitation buffer. Nonetheless, the PLA demonstrated close proximity of the two proteins *in vivo*. Moreover, the need of EGFR for generating the PLA signal supports our model of EGFR being the bridge between STING and Syk (Fig. 11).

STING activation by phosphorylation of two Tyr residues by two receptor-bound Tyr kinases appears to fit a general pattern for the activation of intracellular membrane-bound pattern recognition receptors (PRRs) that recognize nucleic acids. It is reminiscent of Toll-like receptor 3 (TLR3) activation; in that case, EGFR and Src are the relevant kinases (32). We have observed a similar need of two Tyr kinases for the activation of TLR9 signaling by phosphorylation of the receptor (32; our unpublished data). Like TLR3 phosphorylation, phosphorylation of the two Tyr residues of STING happened in a specific sequential manner: Tyr240 phosphorylation preceded Tyr245 phosphorylation. One can speculate that phosphorylation of the first Tyr residue by Syk causes a conformational change of STING to make the second Tyr residue accessible to EGFR. Because ligand-bound STING is a dimer, phosphorylation of STING, EGFR, and Syk probably happens in *trans*; i.e., a kinase bound to one subunit of STING phosphorylates a Tyr residue of the other subunit of STING. Like Tyr245 phosphorylation, phosphorylation of Tyr240 affected translocation of STING from the ER to other compartments. But, whereas without Tyr245 phosphorylation STING did not transit from the Golgi body to the late endosome, in the absence of Tyr240 phosphorylation, STING did not even transit from the ER to the ERGIC, the first compartment in the translocation pathway. The molecular basis of this observation remains to be investigated.

The biological importance of the involvement of Syk in STING signaling was clearly demonstrated by its effect on HSV-1 replication. In cells without STING, inhibiting Syk had no effect on virus yields, indicating that Syk activity was not required *per se* for HSV-1 replication. However, in WT cells, viral activation of STING signaling, as measured by the induction of IFN mRNA, required Syk, and consequently, HSV-1 replicated better in the absence of Syk.

## MATERIALS AND METHODS

### Reagents and antibodies.

2′3′-cGAMP (tlrl-nacga23-02) was purchased from InvivoGen. Ibrutinib (BTK inhibitor; no. S2680), PF-562271 (FAK inhibitor; no. S2890), PP2 (Src inhibitor; no. S7008), saracatinib (Fyn inhibitor; no. S1006), amuvatinib (c-Kit inhibitor; no. S1244), ruxolitinib (Jak1/2 inhibitor; no. S1378), alectinib (ALK inhibitor; no. S2762), gefitinib (EGFR inhibitor; no. S1025), AZD9291 (EGFR inhibitor; no. S7297), and R406 (Syk inhibitor; no. S2194) were obtained from Selleck. Polyethyleneimine (PEI; no. 195444) was purchased from Mpbio, and the transfection procedure was as previously described (16, 33). Lipofectamine 2000 (no. 11668019) was obtained from Invitrogen. Antibodies against EGFR (no. 4267), GFP (no. 2956), phospho-Syk (Tyr525/526) (no. 2710), Syk (no. 13198), tubulin (no. 2146), and actin (no. 3700) were from Cell Signaling, and hemagglutinin (HA) antibody (ab18181) was purchased from Abcam. STING antibody (A3575) and GST (AE001) antibodies were obtained from Abclonal Technology.

### pY240- and pY245-specific antibodies.

pY240 (no. CF1P)- and pY245 (no. CF2P)-specific antibodies were raised by Affinity Biosciences. The synthetic peptides CGIKDRVpYSNSIYE and CVYSNSIpYELLE were used as antigens for injecting New Zealand White rabbits to produce pY240-specific and pY245-specific antibodies, respectively. The antibodies were purified from rabbit serum by positive affinity purification using the phosphopeptides bound to Sepharose and negative affinity purification using the corresponding nonphosphorylated peptides. An enzyme-linked immunosorbent assay (ELISA) was used to test the strengths of the antisera.

### Cell culture and transfection.

HT1080 cells, HEK293T cells, HeLa cells, L929 cells, and STING^−/−^ mouse embryo fibroblasts (MEF) were previously described (18). EGFR KO HeLa cells were a kind gift from the Xiaoxia Li lab (34). HeLa Syk KD cells were made with a pLKO.1 short hairpin RNA (shRNA) lentivirus. The cells were maintained in Dulbecco’s modified Eagle medium (DMEM) containing 10% fetal bovine serum (FBS) and penicillin-streptomycin; 293XL cells were purchased from InvivoGen, and hSTING-GFP and hSTING-GFP mutant 293XL cells were generated as previously described (16). Syk knockdown 293XL cells were generated by lentiviral transduction of human Syk-specific shRNA (35), selected by puromycin and picking of the clones; a nontargeting shRNA (no. SHC002) Mission shRNA vector (Sigma-Aldrich) was used as a control. Lipofectamine 2000 was used for plasmid transfection as per the manufacturer’s protocol. For transient transfection into cells, 2′3′-cGAMP (final concentration, 8 μg/mL) was transfected into cells using polyethyleneimine.

### RNAi-mediated knockdown and SgRNA mediated knockout.

A Mission pLKO.1-puro shRNA plasmid (TRCN0000197242) targeting human Syk and a nontargeting control plasmid (SHC002) were purchased from Sigma (St. Louis, MO, USA). Target cells were transduced with lentivirus in the presence of Polybrene (8 μg/mL) following the manufacturer’s instructions. At 16 h postinfection, the medium was removed, and cells were allowed to recover in complete growth medium for 48 h before using selection medium containing puromycin. Introduction of CRISPR-induced genomic deletion was performed by overnight transduction of subconfluent HT1080 cells (human Syk or human STING) with lentivirus (lentiCRISPRv2 expressing Cas9 endonuclease and the appropriate guide RNA sequence). Human Syk single-guide RNA (sgRNA) sequences were GAAAGAAGTTCGACACGCTC and ACGATCTCATGAATCTGTGC, and the human STING sgRNA sequence was GGTGCCTGATAACCTGAGTA. After transduction, virus-containing medium was replaced with complete medium to allow cellular growth for 48 h, at which time cells were selected with puromycin (Syk deletion) or hygromycin (STING deletion). After drug selection, single-cell clones were screened for maximum Syk or STING deficiency using genomic DNA sequencing and Western blotting.

### Western blotting.

Cells were lysed in lysis buffer of 50 mM Tris-HCl (pH 7.4), 150 mM NaCl, 5 mM EDTA, 1 mM dithiothreitol (DTT), 1 mM phenylmethylsulfonyl fluoride (PMSF), and cocktail (Roche) at 4°C for 15 min and centrifuged at 15,000 rpm at 4°C for 10 min; the supernatant was retained. The cell lysates were resolved by SDS-PAGE or used for immunoprecipitation (IP). The proteins were electrophoresed on SDS-PAGE gels and then transferred to the polyvinylidene difluoride (PVDF) membrane (Bio-Rad). The membranes were kept in 5% skim milk in TBST buffer (150 mM NaCl; Tris, pH 7.4; and 0.1% Tween 20) for 60 min at room temperature and then incubated with the primary antibody at cold room overnight. Western blot experiments were performed via the indicated antibodies and visualized by Super-Signal West Pico chemiluminescent substrate (Pierce Chemical).

### Immunoprecipitation and GFP pulldown.

For immunoprecipitations, cells were lysed in buffer containing 20 mM HEPES (pH 7.5), 150 mM NaCl, 10 mM NaF, 1.5 mM MgCl_2_, 10 mM β-glycerophosphate, 2 mM EGTA, 1 mM Na_3_VO_4_,1% (vol/vol) Triton X-100, 0.2% NP-40, and protease inhibitors (Roche Applied Science, Indianapolis, IN, USA). For the STING dimerization and oligomerization experiments, DTT was excluded from the composition of the cell lysate buffer and loading buffer. The whole-cell lysate was centrifuged for 10 min at 4°C, and the supernatant was used for immunoprecipitation via protein A/G Plus agarose (Santa Cruz) and incubated with 3 μg of the mouse or rabbit monoclonal antibodies overnight at 4°C. For the GFP pulldown, the cell lysate supernatant was incubated with GFP trap beads (Chromotek; gta-20) overnight at 4°C. The related beads were washed six times with IP buffer and boiled in SDS-PAGE buffer for 10 min at 95°C. The samples were separated by SDS-PAGE gel and transferred onto PVDF membranes (Bio-Rad). Western blot experiments were performed with the indicated antibodies and visualized with Super-Signal West Pico chemiluminescent substrate (Pierce Chemical).

### *In vitro* Syk kinase assay and Phos-tag gel analysis.

The Syk kinase buffer contained 50 mM HEPES, 0.01% Tween 20, 10 mM MnCl_2_, 10 mM MgCl_2_, 1 mM EGTA, 2.5 mM DTT, and 0.1 mM ATP, pH 7.4. Recombinant Syk protein was purified from Sf9 insect cells (Biotechne; catalog no. 4594-KS-010); 0.4 μg of Syk and approximately 2 μg of GFP-tagged recombinant STING and mutants were added to the reaction buffer for 60 min at 30°C. All reactions were stopped by adding 5× SDS loading buffer, and mixtures were boiled for 10 min at 95°C for Phos-tag gel-related Western blot (WB) analysis and pY240-specific-antibody-related detection. For analysis of phosphorylation of STING-GFP, Phos-tag (Wako) and MnCl_2_ were added to regular 10% SDS-PAGE gels at the levels recommended by the manufacturer to slow migration of phosphorylated STING. Western blotting was performed normally after soaking the gels in 1 mM EDTA for 10 min (3 times, for a total of 30 min) to remove Mn^2+^. All other steps in this analysis were identical to normal SDS-PAGE and immunoblotting protocols.

### RT-PCR.

For RT-PCR, RNA was isolated using a Roche RNA isolation kit. cDNA was extracted with an ImprompII reverse transcription kit (Promega), and 0.5 ng of cDNA was applied to 384-well plates for real-time PCR using Applied Biosystem’s Power SYBR green PCR mix in a Roche LightCycler 480 II instrument. The levels of 18S rRNA were used for normalization. The specificity was confirmed by analysis of the melting curves of the PCR products. The primer sequences were as follows: m-18S rRNA-F, ATTGACGGAAGGGCACCACCAG; m-18S rRNA-R, CAAATCGCTCCACCAACTAAGAACG; m-IFNβ-F, CTTCTCCGTCATCTCCATAGGG; m-IFNβ-R, CACAGCCCTCTCCATCAACT; m-Ccl20-F, AGAAGCAGCAAGCAACTACG; m-Ccl20-R, ACATCTTCTTGACTCTTAGGC; h-18S rRNA-F, CTACCACATCCAAGGAAGCA; h-18S rRNA-R, TTTTTCGTCACTACCTCCCCG; h-IFNβ-F, CAACTTGCTTGGATTCCTACAAAG; h-IFNβ-R, TATTCAAGCCTCCCATTCAATTG; h-Ccl20-F, AAGTTGTCTGTGTGCGCAAATCC; h-Ccl20-R, CCATTCCAGAAAAGCCACAGTTTT.

### HSV-1 infection and replication.

HSV-1 (KOS) propagation was performed as previously described (36). Subconfluent monolayers of HT1080 and HeLa-M reconstituted cells (1 × 10^6^ cells/well in 6-well plates) were inoculated with HSV-1 (multiplicity of infection [MOI], 5) for 1 h at 37°C in incomplete DMEM. Virus titers were determined in 10-fold serial dilutions on Vero cells by plaque assay as previously reported (37). The viral genome load in the infected cells was measured by quantitative PCR (qPCR) by determining the relative expression levels of the HSV-1 protein ICP0, expressed as the ratio of target ICP0 to 18S rRNA, which were graphed by using GraphPad Prism (version 5.0) software.

### Mass spectrometry.

STING protein was purified from 293XL-hSTING-GFP cells by using GFP trap beads (ChromoTek; gta-20). For protein digestions, the bands were cut from the gels as closely as possible and washed and destained in 50% ethanol, 5% acetic acid. The gel pieces were then dehydrated in acetonitrile, dried in a Speed‐vac, and digested by adding 5 μL (10 ng/μL) of trypsin or chymotrypsin in 50 mM ammonium bicarbonate, followed by incubation overnight. The peptides were extracted into two portions of 30 μL each 50% acetonitrile–5% formic acid. The combined extracts were evaporated to <10 μL in a Speed‐vac and then resuspended in 1% acetic acid to make a final volume of ∼30 μL for LC‐MS analysis. The LC‐MS system was a Thermo Scientific Fusion Lumos Tribrid mass spectrometry system. The high-performance liquid chromatography (HPLC) column was a Dionex 15-cm by 75-μm (inside diameter) Acclaim PepMap C_18_, 2 μm, 100 Å, reversed‐phase capillary chromatography column. Five microliters of the volume of the extract was injected, and the peptides, eluted from the column in acetonitrile–0.1% formic acid gradient at a flow rate of 0.25 μL/min, were introduced into the source of the mass spectrometer online. The microelectrospray ion source was operated at 2.5 kV.

The digest was analyzed in both a survey manner and a targeted manner. The survey experiments were performed using the data‐dependent multitask capability of the instrument, acquiring full-scan mass spectra to determine peptide molecular weights and product ion spectra to determine amino acid sequences in successive instrument scans. The LC‐MS/MS data were searched with the programs Mascot and Sequest against both the full human UniProtKB database and specifically against the sequence of STING. The parameters used in this search include a peptide mass accuracy of 10 ppm, fragment ion mass accuracy of 0.6 Da, carbamidomethylated cysteines as a constant modification, and oxidized methionine and phosphorylation at Y as a dynamic modification. The results were filtered based on Mascot ion scores and Sequest XCorr scores. The positively identified phosphopeptides around Y240 and Y245 were manually validated. The targeted experiments involve the analysis of specific STING peptides around Y240 and Y245. The chromatograms for these peptides were plotted based on known fragmentation patterns, and the peak areas of these chromatograms were used to determine the extent of phosphorylation (38, 39).

### Immunofluorescence staining and confocal microscopy.

Cells were cultured in 4-well chamber slides and transfected with the indicated plasmids via Lipofectamine 2000 for 24 h. The cells were then fixed in 4% paraformaldehyde overnight in a cold room and then permeabilized by phosphate-buffered saline (PBS) with 0.1% Triton X-100 for 10 min at room temperature. The cells were washed four times with PBS and then blocked with 10% bovine serum albumin (BSA) in PBS for 1.5 h at room temperature (RT). Then, cells were incubated with related antibodies overnight in a cold room. After being washed three times with PBS for 10 min each time, the cells were incubated with goat anti-rabbit Alexa Fluor 594 (Invitrogen), anti-mouse Alexa Fluor 594 (Invitrogen), or anti-rabbit Alexa Fluor 647 (Invitrogen). Objects were mounted using Vectashield/DAPI (4′,6-diamidino-2-phenylindole), and images were taken by confocal laser scanning microscopy (Leica TCS SP8). Images were processed with Leica LCS software. The Fiji (ImageJ) software colocalization plug-in was used for determining colocalization of two or three proteins, where white dots represent colocalization; for each figure, we set higher parameters in the plug-in software to get clear results, and the control and negative groups do not appear as white spots. Images were photographed at random positions for each condition.

### PLA.

Adherent HeLa cells on the glass slides were transfected with the indicated plasmids. After fixation, cells were pretreated and blocked with 5% BSA. Primary antibodies against GFP and Syk were used for the slides. The secondary antibodies, which were conjugated with oligonucleotides (PLA probe MINUS [Sigma no. DUO92004] and PLA probe PLUS [Sigma no. DUO92002]), were added to the glass slides for 1 h at 37°C. After incubation, a system consisting of two oligonucleotides and a ligase were added to the system for 30 min at 37°C. If the oligonucleotide probes were in close proximity, the ligation reaction between them formed a closed DNA circle. Then, the slides were incubated for amplification by the PLA probe (Sigma no. DUO92008) and the polymerase for 100 min at 37°C. The amplification reaction results in a signal which was seen as a unique fluorescent spot under confocal microscopy. The PLA dots were counted via Fiji software and analyzed as previously described (40).

### Quantification of Western blots and calculation of the extent of Y240 phosphorylation.

ImageJ software was used to quantify the intensities of protein bands on Western blots. The band intensities of STING dimers and oligomers were determined by the software and then divided by values of STING from normal SDS-PAGE gels for normalization. pY240 level was determined by the software and then divided by STING values for normalization. LC-MS/MS analysis showed that 0.7% of STING Y240 was phosphorylated after cGAMP treatment of 293XL STING-GFP cells. pY240 was also quantified in the same sample using the pY240-specific antibody in Western blots; the conversion factor allowed us to convert pY240 band intensities to percent phosphorylation in other samples.

### Statistical analysis.

The statistical significance of differences between the two groups was tested via a two-tailed *t* test using Prism 5 software (GraphPad).
